# Supplementary material for: Anoxia activates CRISPR–Cas immunity in the mouse intestine
Source: Nat Microbiol. 2025 Oct 29;10(12):3069–74. doi: 10.1038/s41564-025-02172-8 (PMC12669028; doi:10.1038/s41564-025-02172-8)
Supplement: Supplementary file 1 — Reporting Summary [file 41564_2025_2172_MOESM1_ESM.pdf]

# nature portfolio

Corresponding author(s): Ian W. Campbell
Matthew K. Waldor

Last updated by author(s): Sep 25, 2025

# Reporting Summary

Nature Portfolio wishes to improve the reproducibility of the work that we publish. This form provides structure for consistency and transparency in reporting. For further information on Nature Portfolio policies, see our Editorial Policies and the Editorial Policy Checklist.

## Statistics

For all statistical analyses, confirm that the following items are present in the figure legend, table legend, main text, or Methods section.

| n/a | Confirmed | |
|---|---|---|
| ☐ | ☒ | The exact sample size (*n*) for each experimental group/condition, given as a discrete number and unit of measurement |
| ☐ | ☒ | A statement on whether measurements were taken from distinct samples or whether the same sample was measured repeatedly |
| ☐ | ☒ | The statistical test(s) used AND whether they are one- or two-sided<br>*Only common tests should be described solely by name; describe more complex techniques in the Methods section.* |
| ☒ | ☐ | A description of all covariates tested |
| ☐ | ☒ | A description of any assumptions or corrections, such as tests of normality and adjustment for multiple comparisons |
| ☐ | ☒ | A full description of the statistical parameters including central tendency (e.g. means) or other basic estimates (e.g. regression coefficient) AND variation (e.g. standard deviation) or associated estimates of uncertainty (e.g. confidence intervals) |
| ☐ | ☒ | For null hypothesis testing, the test statistic (e.g. *F*, *t*, *r*) with confidence intervals, effect sizes, degrees of freedom and *P* value noted<br>*Give P values as exact values whenever suitable.* |
| ☒ | ☐ | For Bayesian analysis, information on the choice of priors and Markov chain Monte Carlo settings |
| ☒ | ☐ | For hierarchical and complex designs, identification of the appropriate level for tests and full reporting of outcomes |
| ☒ | ☐ | Estimates of effect sizes (e.g. Cohen's *d*, Pearson's *r*), indicating how they were calculated |

*Our web collection on statistics for biologists contains articles on many of the points above.*

## Software and code

Policy information about availability of computer code

| | |
|---|---|
| Data collection | Transposon insertion sequencing data were collected using an Illumina NextSeq 1000. RNA-sequencing data were collected with an Illumina NovaSeq X Plus. Sequencing data demultiplexing, quality control, and adapter trimmer were performed with Illumina bcl convert. Quantitative PCR was performed using an Applied Biosystems StepOnePlus Real-Time PCR System. |
| Data analysis | Data analysis was performed using CLC genomics workbench (version 24.0.1), GraphPad Prism (version 10.4.1), Python (version 3.12), and Microsoft Excel. Custom scripts for Tn-seq analysis were originally developed in Basta & Campbell et. al., 2025 and are deposited online at https://github.com/dbasta27/InducTn-seq. |

For manuscripts utilizing custom algorithms or software that are central to the research but not yet described in published literature, software must be made available to editors and reviewers. We strongly encourage code deposition in a community repository (e.g. GitHub). See the Nature Portfolio guidelines for submitting code & software for further information.

## Data

Policy information about availability of data

All manuscripts must include a data availability statement. This statement should provide the following information, where applicable:

- Accession codes, unique identifiers, or web links for publicly available datasets
- A description of any restrictions on data availability
- For clinical datasets or third party data, please ensure that the statement adheres to our policy

RNA-seq and InducTn-seq sequencing reads are deposited in the Sequencing Read Archive (SRA) under accession no. PRJNA1254768. Reads were mapped to the C. rodentium ICC168 genome FN543502.1. Source data are provided with this paper as a source data file.

## Research involving human participants, their data, or biological material

Policy information about studies with human participants or human data. See also policy information about sex, gender (identity/presentation), and sexual orientation and race, ethnicity and racism.

| | |
|---|---|
| Reporting on sex and gender | N/A |
| Reporting on race, ethnicity, or other socially relevant groupings | N/A |
| Population characteristics | N/A |
| Recruitment | N/A |
| Ethics oversight | N/A |

Note that full information on the approval of the study protocol must also be provided in the manuscript.

# Field-specific reporting

Please select the one below that is the best fit for your research. If you are not sure, read the appropriate sections before making your selection.

☒ Life sciences ☐ Behavioural & social sciences ☐ Ecological, evolutionary & environmental sciences

For a reference copy of the document with all sections, see nature.com/documents/nr-reporting-summary-flat.pdf

# Life sciences study design

All studies must disclose on these points even when the disclosure is negative.

| | |
|---|---|
| Sample size | Sample sizes were determined by the variation observed in previous studies and preliminary experiments. |
| Data exclusions | No data were excluded from this study. |
| Replication | All attempts at replication were successful. Replicates are described in figure legends. |
| Randomization | Cages of animals were randomly assigned to experimental groups. Animals were not randomized within a cage due to the possibility of transmission between mice. |
| Blinding | Blinding was not used in these studies because the quantifiable variables (RNA-sequencing and plasmid exclusion) are not subject to investigator bias. |

# Reporting for specific materials, systems and methods

We require information from authors about some types of materials, experimental systems and methods used in many studies. Here, indicate whether each material, system or method listed is relevant to your study. If you are not sure if a list item applies to your research, read the appropriate section before selecting a response.

## Materials & experimental systems

| n/a | Involved in the study |
| --- | --- |
| ☒ | ☐ Antibodies |
| ☒ | ☐ Eukaryotic cell lines |
| ☒ | ☐ Palaeontology and archaeology |
| ☐ | ☒ Animals and other organisms |
| ☒ | ☐ Clinical data |
| ☒ | ☐ Dual use research of concern |
| ☒ | ☐ Plants |

## Methods

| n/a | Involved in the study |
| --- | --- |
| ☒ | ☐ ChIP-seq |
| ☒ | ☐ Flow cytometry |
| ☒ | ☐ MRI-based neuroimaging |

# Animals and other research organisms

Policy information about studies involving animals; ARRIVE guidelines recommended for reporting animal research, and Sex and Gender in Research

| | |
| --- | --- |
| Laboratory animals | Adult (9–12 weeks) C57BL/6J mice were purchased from Jackson Laboratory (strain #000664). |
| Wild animals | No wild animals were used in this study. |
| Reporting on sex | RNA-sequencing was performed on a single group of 3 female mice. RNA-sequencing data were confirmed and expanded upon with a plasmid exclusion assay, which was performed using an equal mix of male and female mice. No difference was observed between sexes and disaggregated data will be included in source data. |
| Field-collected samples | No field-collected samples were used in this study. |
| Ethics oversight | Animal studies were conducted at Brigham and Women's Hospital in compliance with the 'Guide for the Care and Use of Laboratory Animals' and according to protocols reviewed and approved by the Brigham and Women's Hospital's Institutional Animal Care and Use Committee under protocol 2016N000416. |

Note that full information on the approval of the study protocol must also be provided in the manuscript.

# Plants

| | |
| --- | --- |
| Seed stocks | N/A |
| Novel plant genotypes | N/A |
| Authentication | N/A |
